# Emerging Paramyxoviruses: Receptor Tropism and Zoonotic Potential

**DOI:** 10.1371/journal.ppat.1005390

**Published:** 2016-02-25

**Authors:** Antra Zeltina, Thomas A. Bowden, Benhur Lee

**Affiliations:** 1 Division of Structural Biology, Wellcome Trust Centre for Human Genetics, University of Oxford, Oxford, United Kingdom; 2 Icahn School of Medicine at Mount Sinai, New York, New York, United States of America; University of Kentucky, Lexington, UNITED STATES

## Introduction

Emerging infectious disease (EID) events are dominated by zoonoses: infections that are naturally transmissible from animals to humans or vice versa [1]. A worldwide survey of ~5,000 bat specimens identified 66 novel paramyxovirus species—more than double the existing total within this family of viruses [2]. Also, novel paramyxoviruses are continuously being discovered in other species, such as rodents [3–5], shrews [6], wild and captivated reptiles [7], and farmed fish [8], as well as in domestic cats [9] and horses [10]. Paramyxoviruses exhibit one of the highest rates of cross-species transmission among RNA viruses [11], and paramyxoviral infection in humans can cause a wide variety of often deadly diseases. Thus, it is important to understand the determinants of cross-species transmission and the risk that such events pose to human health. Whilst pathogen diversity and human encroachment play important roles, here, we focus on receptor tropism and envelope determinants for zoonosis of emerging paramyxoviruses.

## Limitations of Conventional Sequence-Based Phylogenetic Analysis

**Fig 1:**
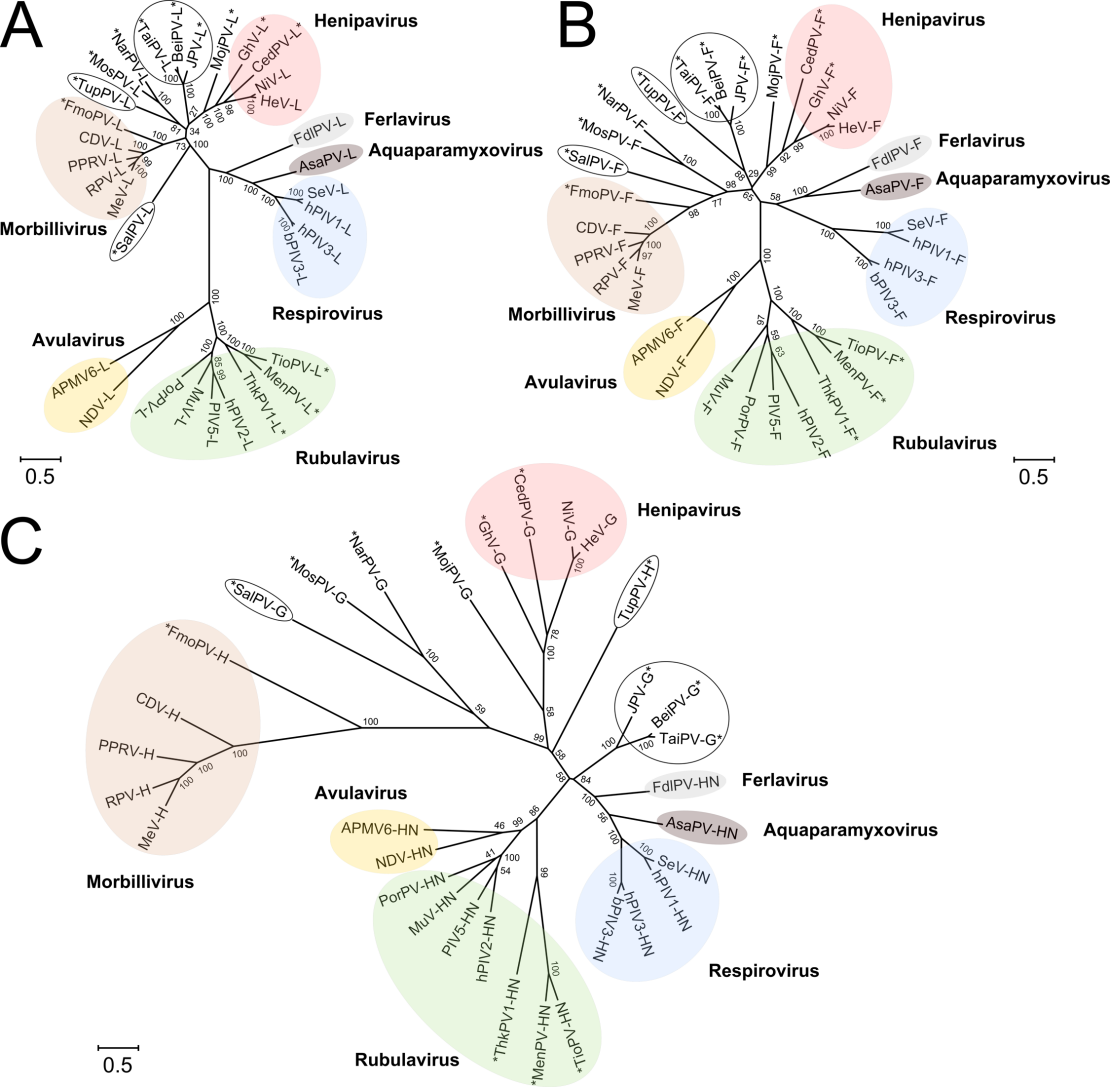
**Maximum likelihood phylogenies of Paramyxoviridae L (A), F (B), and HN/H/G (C) protein sequences using MEGA6 (Molecular Evolutionary Genetics Analysis Version 6.0) [34], based on the LG+G+I+F model [35].** Scale bar indicates amino acid substitutions per site. Numbers at the nodes represent bootstrap values (1,000 replicates). Colored circles are used to indicate genera and empty black circles indicate viruses with varying positions between the L, F, and HN/H/G phylogenies. Uncategorized viruses awaiting taxonomic evaluation by the International Committee on Taxonomy of Viruses are marked with an asterisk. Virus names (abbreviations) and GenBank accession numbers are as follows: Mojiang virus (MojPV) NC_025352; Ghanaian bat henipavirus (GhV) HQ660129; Cedar virus (CedPV) NC_025351; Nipah virus (NiV) NC_002728; Hendra virus (HeV) NC_001906; Sendai virus (SeV) NC_001552; human parainfluenza virus 1 (hPIV1) NC_003461; human parainfluenza virus 3 (hPIV3) AY283063 (for HN) and NC_001796.2 (for L and F); bovine parainfluenza virus 3 (bPIV3) NC_002161; Tuhoko virus 1 (ThkPV1) NC_025410; Menangle virus (MenPV) NC_007620; Tioman virus (TioPV) NC_004074; parainfluenza virus 5 (PIV5) NC_006430; human parainfluenza virus 2 (hPIV2) NC_003443; porcine rubulavirus (PorPV) NC_009640; mumps virus (MuV) NC_002200; avian paramyxovirus 6 (APMV6) NC_003043; Newcastle disease virus (NDV) AF212323 (for HN) and NC_002617 (for L and F); canine distemper virus (CDV) AY386315; peste-des-petits-ruminants virus (PPRV) FJ905304; rinderpest virus (RPV) JN234010; measles virus (MeV) NC_001498; Mossman virus (MosPV) NC_005339; Nariva virus (NarPV) NC_017937.1; Beilong virus (BeiPV) NC_007803; Tailam virus (TaiPV) NC_025355; J paramyxovirus (JPV) NC_007454; Salem virus (SalPV) NC_025386; Fer-de-lance virus (FdlPV) NC_005084; Atlantic salmon paramyxovirus (AsaPV) NC_025360; Tupaia paramyxovirus (TupPV) NC_002199; feline morbillivirus (FmoPV) JQ411014.

The Paramyxoviridae family is divided into two subfamilies, Paramyxovirinae and Pneumovirinae. The subfamily Paramyxovirinae is currently classified into seven genera (Fig 1) (http://www.ictvonline.org/virusTaxonomy.asp). Because of conservation of sequence and functionality, it has been proposed that phylogenetic analysis of large polymerase (L) (Fig 1A), fusion (F) (Fig 1B), and matrix (M) protein sequences should be used for classification of paramyxoviruses [12]. However, whilst this classification method is useful for assignment of novel paramyxoviruses, it has a limited capacity for assessing which viruses have zoonotic potential and are relevant to human health. It is interesting to note that if an analogous phylogenetic analysis is performed on the viral attachment protein, placement of as-yet-unclassified viruses does vary (Fig 1C), a phenomenon attributable to the greater level of genetic diversity in the attachment glycoprotein with respect to L, F, and M proteins. The observed differential levels of sequence variation may be rationalized by considering the function of these proteins: whilst sequence diversification in the attachment protein enables evasion of the host immune response and varied utilization of host cellular receptors, paramyxoviral L, F, and M proteins are more conserved in function (i.e., responsible for replication, membrane fusion, and budding, respectively) and thus are subject to more stringent evolutionary constraints.

## Zoonotic Paramyxoviruses: Past, Present, and Future

Zoonotic spillovers have been observed across several paramyxovirus genera. For example, Newcastle disease virus (NDV), a major poultry pathogen, can cause occasional conjunctivitis and influenza-like symptoms in humans [13]. Measles virus (MeV), now considered a strictly human pathogen, may have originated from the common ancestor of the recently eradicated cattle pathogen, rinderpest virus (RPV), and caused disease in humans around the 11th and 12th centuries [14]. Over the last decades, two highly pathogenic henipaviruses, Nipah virus (NiV) and Hendra virus (HeV), emerged from fruit bats in Asia and Australia, causing severe disease in humans. NiV and HeV infections result in respiratory and encephalitic illness, with mortality ranging between 50% to 100% [15]. Moreover, Mojiang paramyxovirus (MojPV), a henipavirus-like virus, has been implicated in the deaths of three miners in China in 2012, following potential zoonotic transmission from rats [16]. The recent identification of novel henipaviruses and rubulaviruses of unknown pathogenicity in African bats [2] and the serological detection of African henipaviruses and rubulaviruses in humans [17,18] underscores the near-global threat of these pathogens.

In addition, even long-known animal paramyxoviruses may pose a threat to human health. For example, it has been suggested that following MeV eradication, MeV vaccinations may be curbed, and the close morbillivirus relative, canine distemper virus (CDV), which resides in a number of mammalian hosts, including wolves, foxes, and dogs, might emerge as a new human pathogen. Indeed, it has become apparent over the last decades that the host range of CDV extends beyond the long-known and well-established hosts, with infections being observed in seals, lions, and monkeys [19].

## Envelope Determinants of Paramyxoviral Host Tropism

Factors influencing paramyxoviral host tropism and virulence affect almost every stage of the virus lifecycle, including host cell entry, viral assembly, budding, and immune antagonism or evasion. In the context of viral entry, pathogenicity can be limited by the requirement for cleavage of the paramyxoviral fusion protein precursor, F_0_, by cellular proteases to a mature fusion protein, F. While most paramyxoviral F_0_ are cleaved by ubiquitous proteases such as furin or cathepsin L, murine Sendai virus (SeV) F_0_ is only susceptible to trypsin-like proteases [20]. As a consequence of the limited tissue distribution of these enzymes and the exclusively apical budding of SeV, infection remains localized in the respiratory tract [21].

**Fig 2:**
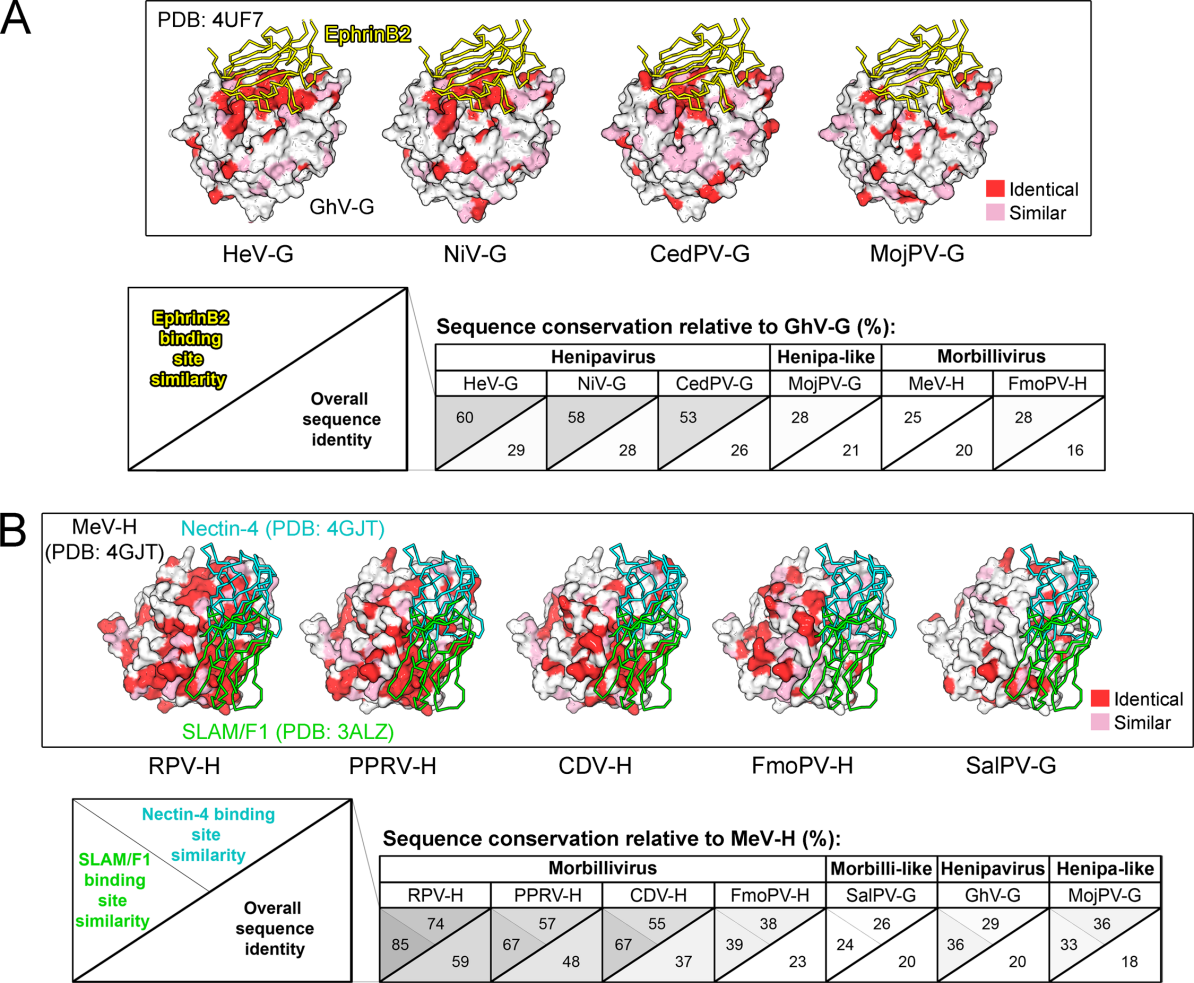
Mapping sequence conservation onto henipavirus and morbillivirus attachment glycoproteins. (A) Crystal structure of Ghanaian bat henipavirus attachment glycoprotein (GhV-G, PDB [Protein Data Bank] ID 4UF7) in complex with ephrinB2. EphrinB2 is shown as a yellow ribbon and GhV-G is shown in surface representation and colored according to sequence conservation with (left to right) HeV-G, NiV-G, CedPV-G, and MojPV-G. Sequence identical residues are colored red and similar residues pink. At the bottom of the panel are tables summarizing overall glycoprotein sequence identity (bottom right) and sequence similarity at the ephrin receptor-binding site (top left), with respect to GhV-G. (B) Crystal structure of measles virus hemagglutinin (MeV-H) in complex with nectin-4 cell surface receptor (PDB ID 4GJT), with the position of SLAM/F1 (green ribbon) receptor-binding shown (based upon structural overlay with the MeV-H-SLAM/F1 crystal structure; PDB ID 3ALZ). Nectin-4 and SLAM/F1 are shown as cyan and green ribbons, respectively. MeV-H is shown in surface representation and colored according to sequence conservation, as in panel A, with RPV-H, PPRV-H, CDV-H, FmoPV-H, and SalPV-G (left to right). At the bottom of the panel are tables summarizing overall glycoprotein sequence identity (bottom right) and sequence similarity at the nectin-4– (top) and SLAM/F1-binding sites (left), with respect to MeV-H. The tables are color-coded from dark gray (highly conserved) to white (variable).

The capacity of an attachment glycoprotein to specifically target receptors expressed on the host cell surface is another crucial determinant of paramyxoviral host tropism [22,23]. Paramyxovirus attachment glycoproteins are type-II membrane proteins composed of an N-terminal cytoplasmic and transmembrane region, a stalk domain, and a receptor-binding domain. The receptor-binding domain forms a six-bladed β-propeller fold and is the key determinant of host receptor specificity. Thus far, paramyxovirus attachment glycoproteins have been divided into three major groups: hemagglutinin (H), hemagglutinin-neuraminidase (HN), and attachment glycoproteins (G) (Fig 1C). Despite the common fold of the receptor attachment domain, the overall sequence conservation is low (Fig 2). This reflects the structural plasticity of the β-propeller scaffold and its ability to adapt to different host cell receptors and receptor engagement modes (Fig 2) [23]. Cell surface receptors utilized by paramyxoviruses can be either protein or carbohydrate. HN glycoproteins encode a structurally well-conserved binding motif [24,25], which recognizes sialic acid (N-acetylneuraminic acid), a terminal saccharide present on cellular glycoproteins and glycolipids. Because of the abundance of sialic acid at the cell surface of vertebrates, it is likely that factors other than receptor specificity may play a major role in the host tropism of HN-bearing viruses. H and G glycoproteins, on the other hand, recognize proteinaceous cell surface receptors, such as SLAM/F1 (signaling lymphocytic activation molecule family member 1, CD150) and nectin-4 for morbilliviruses and ephrinB2 and ephrinB3 for NiV and HeV [20,23]. Unlike the glycan receptors of HN-displaying paramyxoviruses, the conservation and tissue distribution of proteinaceous receptors utilized by henipaviruses and morbilliviruses are thought to play a major role in determining host and cellular tropism.

Amongst Paramyxoviridae family members, henipaviruses exhibit a remarkably broad host range, with natural infections observed in bats, horses, pigs, cats, dogs, goats, and humans. The usage of the cell surface receptor ephrinB2, which is highly conserved across vertebrate species [26], is one critical determinant of the wide host tropism. Moreover, the expression of ephrinB2 on microvascular endothelial cells [27], neurons [26], and respiratory epithelium [28] provides a molecular rationale for the efficient systemic dissemination of henipaviruses [26] and the organ-specific symptomology that they cause. The spread of morbilliviruses, such as MeV and CDV, on the other hand, is achieved by primary infection of airway dendritic cells and/or alveolar macrophages via SLAM/F1. Following viral amplification in lymphatic organs, morbilliviruses migrate back into airways via basal-lateral infection of epithelial cells using the adherens junction molecule nectin-4 (also known as PVRL4, poliovirus receptor-related 4), allowing transmission to new susceptible hosts [29]. Although adaption to new proteinaceous receptors is not the sole determinant of cross-species infection, it is interesting to note that, under experimental cell culture conditions, CDV requires no adaptive alteration in the H attachment glycoprotein to utilize human nectin-4, and only a single amino acid change is necessary to adapt to human SLAM/F1 [19].

## Henipavirus and Morbillivirus Attachment Glycoproteins as Tropism Predictors of Emerging Paramyxoviruses

Within the last two decades, crystal structures of receptor attachment domains from several biomedically important paramyxoviruses, including MeV, NiV, and HeV, in complex with their functional cell-surface receptors, have revealed the protein interaction interfaces utilized for receptor engagement [23,30]. Using this information, it becomes possible to make predictions for whether receptor specificity is conserved for newly emergent paramyxoviruses. For example, mapping of sequence similarity between the G protein of the Ghanaian bat henipavirus (GhV) (see note in [31]), an emergent African paramyxovirus isolated from bats, onto the crystal structure of NiV-G complexed with ephrinB2 revealed an elevated level of conservation at the receptor-binding site, suggesting that GhV-G and NiV-G share ephrinB2 as an entry receptor [17]. The recent crystal structure of GhV-G in complex with the ephrinB2 confirmed this prediction [32].

If we extend this analysis to include viruses within or peripherally related to protein-binding henipavirus and morbillivirus genera (Fig 2), well-established henipaviruses (NiV-G, HeV-G, CedV-G) and morbilliviruses (RPV-H, PPRV-H, and CDV-H) show a high level of sequence conservation at their respective receptor-binding sites; in contrast, viruses more peripherally related to both of these respective genera lack this conservation (Fig 2). For example, the henipavirus-like, rodent-borne MojPV [16] lacks significant sequence conservation at the ephrin receptor-binding surfaces of GhV-G (Fig 2A). Considering that the henipavirus cell surface receptor ephrinB2 is highly conserved across vertebrate species [33], it seems unlikely that the yet structurally and functionally uncharacterized MojPV will recognize the receptor used by well-established henipaviruses. Likewise, the attachment glycoproteins from morbilli-like Salem virus (SalPV), recently isolated from horses [10], and the putatively assigned feline morbillivirus (FmoPV) [9] lack significant sequence conservation at the SLAM/F1 and nectin-4 receptor-binding surfaces of MeV-H (Fig 2B) and are thus are less likely to recognize *human* SLAM/F1 and nectin-4 receptors.

Sequence conservation of cellular receptors is an additional parameter to consider when predicting viral tropism, especially given that the presented predictive strategy solely utilizes crystal structures of primate receptor complexes. Unlike ephrinB2 and nectin-4, SLAM/F1 exhibits a relatively lower level of sequence conservation between species (e.g., human and feline SLAM/F1 receptors exhibit 66% sequence identity at the amino acid level). Thus, whilst our receptor-binding site conservation analysis may be useful for predicting the capacity of emerging viruses to utilize the well-conserved ephrinB2 and nectin-4 receptors of any vertebrate species, the sequence divergence of the SLAM/F1 receptor is likely to limit the predictive power of SLAM/F1 usage in vertebrate species other than primates. This is an important consideration given that both SLAM/F1 and nectin-4 are tissue-specific receptors, and both would most likely be required to be utilized for productive cross-species transmission.

## Re-evaluation of How to Define Genera in the Paramyxoviridae

As a result of improved and more rigorous viral surveillance, the number of new and unclassified paramyxoviruses has grown enormously over the past few years. For example, even with the recent addition of two paramyxovirus genera, *Aquaparamyxovirus* and *Ferlavirus*, many recently identified viruses remain as-yet-unclassified (Fig 1). Whilst many of these viruses have not yet been isolated and fully characterized, their discovery enhances our appreciation of paramyxoviral diversity. Furthermore, the addition of these new viruses poses a challenge with regards to classification and taxonomy, specifically in the context of drawing and defining boundaries of paramyxoviral genera. We suggest that the process of inferring viral boundaries, which uses traditional phylogeny-based calculations as a base, could incorporate host tropism data and structurally-guided analyses to aid in the finer definitions.

The incorporation of such analyses may also be used to re-evaluate previous phylogenetic analysis-based predictions. For example, MojPV has been putatively classified as a henipavirus. However, upon mapping sequence conservation between GhV-G and MojPV-G onto the GhV-G-ephrinB2 co-crystal structure, it becomes apparent that MojPV is unlikely to utilize ephrinB2 receptor (Fig 2A). Indeed, in light of the expanding viral universe, it is possible that the absence of conserved receptor tropism and pathobiology may justify re-evaluation of existing genera boundaries to reflect both the conservation of sequence and function.
